# Supplementary material for: Prognostic Role of Inflammatory and Nutritional Biomarkers in Non-Small-Cell Lung Cancer Patients Treated with Immune Checkpoint Inhibitors Alone or in Combination with Chemotherapy as First-Line
Source: Cancers (Basel). 2024 Nov 19;16(22):3871. doi: 10.3390/cancers16223871 (PMC11592697; doi:10.3390/cancers16223871)
Supplement: Supplementary file 1 [file cancers-16-03871-s001.zip › cancers-3322942-supplementary.pdf]

Table S1: Patients' characteristics.

		All	ICI	ICI + chemo	p value
		**n 100 %**	**n (%)**	**n (%)**	
		191 (100)	93	98	
**ALK**	**wild type**	158 (82.7)	74 (79.6)	84 (85.7)	0.339
	**not determinable**	1 (0.5)	1 (1.1)	0 (0)	
	**unknown**	32 (16.8)	18 (19.4)	14 (14.3)	
**Biom diagnosis**	**sequenom**	112 (58.6)	66 (71.0)	46 (46.9)	<0.001
	**RT-PCR**	4 (2.1)	3 (3.2)	1 (1.0)	
	**NGS**	45 (23.6)	8 (8.6)	37 (37.8)	
	**unknown**	30 (15.7)	16 (17.2)	14 (14.3)	
**EGFR**	**wildt type**	156 (81.7)	75 (80.6)	81 (82.7)	0.666
	**mutant**	1 (0.5)	0 (0)	1 (1.0)	
	**not determinable**	3 (1.6)	1 (1.1)	2 (2.0)	
	**unknown**	31 (16.2)	17 (18.3)	14 (14.3)	
**KRAS**	**wild type**	96 (50.3)	38 (40.9)	58 (59.2)	0.042
	**mutant**	62 (32.5)	36 (38.7)	26 (26.5)	
	**unknown**	33 (17.3)	19 (20.4)	14 (14.3)	
**other_mut**	**no**	124 (64.9)	62 (66.7)	62 (63.3)	0.176
	**yes**	34 (17.8)	12 (12.9)	22 (22.4)	
	**unknown**	33 (17.3)	19 (20.4)	14 (14.3)	
**PLR cat**	**≤180**	96 (50.3)	55 (59.1)	41 (41.8)	0.021
	**>180**	95 (49.7)	38 (40.9)	57 (58.2)	
**PNI cat**	**<45**	112 (58.6)	48 (51.6)	64 (65.3)	0.058
	**≥45**	79 (41.4)	45 (48.4)	34 (34.7)	
**ROS1**	**wild type**	158 (82.7)	74 (79.6)	84 (85.7)	0.339
	**not determinable**	1 (0.5)	1 (1.1)	0 (0)	
	**unknown**	32 (16.8)	18 (19.4)	14 (14.3)	
**Visceral mets**	**no**	67 (35.1)	37 (39.8)	30 (30.6)	0.225
	**yes**	124 (64.9)	56 (60.2)	68 (69.4)	
		**Median (IQ range)**			
**Ca**		9.30 (9.00, 9.60)	9.40 (9.10, 9.60)	9.30 (9.00, 9.50)	0.258
**dNLR**		2.40 (1.73, 3.15)	2.39 (1.76, 2.93)	2.41 (1.69, 3.40)	0.515
**Eos**		200.00 (100.00, 300.00)	200.00 (100.00, 400.00)	100.00 (0.00, 200.00)	<0.001
**H_cm**		1.68 (1.63, 1.75)	1.68 (1.65, 1.75)	1.68 (1.63, 1.75)	0.570
**Hb**		13.20 (11.95, 14.55)	13.30 (12.20, 14.60)	13.05 (11.75, 14.50)	0.603

**Height**		168.00 (163.50, 175.00)	168.00 (165.00, 175.00)	168.00 (163.00, 174.75)	0.570
**LDH**		215.00 (172.50, 277.50)	215.00 (172.00, 257.00)	214.00 (174.25, 293.50)	0.368
**Linf**		1500.00 (1100.00, 2100.00)	1600.00 (1100.00, 2200.00)	1500.00 (1100.00, 2000.00)	0.257
**Neut**		6200.00 (4600.00, 8400.00)	6200.00 (4900.00, 9000.00)	6350.00 (4300.00, 8075.00)	0.357
**NLR**		3.90 (2.65, 6.06)	3.86 (2.79, 5.93)	3.97 (2.50, 6.50)	0.831
**PLR**		179.41 (132.14, 275.27)	163.70 (130.71, 260.91)	201.83 (133.19, 300.69)	0.077
**PLT**		287.00 (221.00, 353.00)	280.00 (222.00, 338.00)	295.00 (218.25, 359.75)	0.553
**PNI**		43.00 (37.25, 47.50)	44.00 (38.50, 48.50)	42.00 (36.12, 47.00)	0.079
**SII**		1179947.37 (688510.00, 1987687.50)	1094333.33 (763200.00, 1720000.00)	1269254.90 (626325.00, 2410400.00)	0.574
**Weight**		69.00 (60.00, 80.00)	70.00 (61.00, 78.00)	69.00 (60.00, 80.00)	0.788
**White**		9000.00 (6850.00, 11200.00)	9100.00 (7400.00, 11200.00)	8700.00 (6525.00, 11075.00)	0.183
**Metastatic sites**		2 (1,3)	2 (1,3)	2 (1,3)	0.309
**ALI score**		21.68 (11.88, 34.91)	22.68 (12.40, 33.59)	21.34 (11.68, 36.15)	0.828
**BMI**		24.16 (21.92, 26.83)	24.31 (21.95, 26.81)	23.78 (21.91, 26.83)	0.993
**dNLR**	**≤3**	138 (72.3)	72 (77.4)	66 (67.3)	0.146
	**>3**	53 (27.7)	21 (22.6)	32 (32.7)	
**NLR**	**≤5**	121 (63.4)	57 (61.3)	64 (65.3)	0.652
	**>5**	70 (36.6)	36 (38.7)	34 (34.7)	
**Response**	**PR**	74 (38.7)	37 (39.8)	37 (37.8)	0.151
	**SD**	39 (20.4)	13 (14.0)	26 (26.5)	
	**CR**	4 (2.1)	3 (3.2)	1 (1.0)	
	**PD**	57 (29.8)	29 (31.2)	28 (28.6)	
	**NA (not assessable)**	17 (8.9)	11 (11.8)	6 (6.1)	
**RT**	**no**	132 (69.1)	62 (66.7)	70 (71.4)	0.532
	**yes**	59 (30.9)	31 (33.3)	28 (28.6)	
**Schedule**	**pembro**	81 (42.4)	81 (87.1)	0 (0)	<0.001
	**pembro+carbo+pem**	61 (31.9)	0 (0)	61 (62.2)	
	**pembro+cis+pem**	22 (11.5)	0 (0)	22 (22.4)	
	**pembro+carbo+pacl**	14 (7.3)	0 (0)	14 (14.3)	
	**atezo**	12 (6.3)	12 (12.9)	0 (0)	
	**chemo+ipi+nivo**	1 (0.5)	0 (0)	1 (1.0)	
